# The association of alcohol with circulating total fibrinogen and plasma clot density is mediated by *fibrinogen* and *FXIII* genotypes

**DOI:** 10.1186/s12959-020-00249-4

**Published:** 2020-11-30

**Authors:** Petro Hannie Rautenbach, Cornelie Nienaber-Rousseau, Marlien Pieters

**Affiliations:** 1 Centre of Excellence for Nutrition, North-West University, Private bag x6001, Nutrition, Box 594, Potchefstroom, 2520 South Africa; 2 Medical Research Council Unit for Hypertension and Cardiovascular Disease, North-West University, Potchefstroom, South Africa

**Keywords:** ALT, AST, Coagulation, Gamma-glutamyltransferase, Percentage carbohydrate deficient transferrin, Self-reported alcohol intake

## Abstract

**Background:**

Alcohol consumption is associated with haemostasis and so may influence cardiovascular conditions. It is unknown whether the association of alcohol with total and γ’ fibrinogen concentrations, as well as clot structure, are modulated by fibrinogen and *factor (F) XIII* single nucleotide polymorphisms (SNPs).

**Methods:**

Total fibrinogen, γ’ fibrinogen and clot properties of 2010 healthy Africans residing in South Africa were measured in relation to alcohol intake as well as its markers – gamma-glutamyltransferase (GGT), percentage carbohydrate deficient transferrin (%CDT), aspartate aminotransferase (AST), and alanine aminotransferase (ALT). Fourteen *fibrinogen* and two SNPs in the *FXIII* gene were genotyped to determine their influence.

**Results:**

Alcohol intake and its markers correlated negatively with fibrinogen and clot lysis time (CLT) as well as with most of the clot properties. Percentage γ’ fibrinogen correlated positively with AST and negatively with alcohol intake. We then stratified for alcohol intake and found inverse associations between γ’ fibrinogen and both %CDT and GGT–CDT in consumers, but the positive association with AST remained only in abstainers. Alcohol intake and its markers modulated the influence of *fibrinogen* SNPs on total fibrinogen concentrations and the *fibrinogen* SNPs as well as an *FXIII* SNP on clot density (all *p* < 0.004).

**Conclusion/s:**

We show for the first time that some individuals harbour certain genotypes that, in combination with alcohol consumption, might predispose or protect them from haemostatic factors that might lead to the development of cardiovascular disease. Studies are needed to clarify the mechanisms related to the interplay between alcohol and the gene variants observed here.

## Background

The burgeoning increase in cardiovascular disease (CVD) is a global concern [1]. In many cases cardiovascular conditions can be adversely affected, but may also be prevented or improved by behaviour [2]. Alcohol intake is one such factor that influence CVD risk – consumed in light to moderate amounts risk decreases [3], whereas binge drinking increases it [4]. Alcohol can alter CVD risk through haemostatic processes [5].

The circulating glycoprotein fibrinogen [6, 7], and its splice variant γ’ fibrinogen [8], that provide the main structure for blood clots; as well as factor XIII (FXIII) that stabilises the formation of clots and clot structure [9] have been implicated in CVD [10]. Moderate alcohol consumption reduces total fibrinogen concentrations [11–14]. However, whether alcohol intake influences γ’ fibrinogen is unknown. Moreover, the impact of alcohol on the properties of blood clots is complex and also remains to be elucidated [15].

Single nucleotide polymorphisms (SNPs) within the *fibrinogen* *α*, *β* and *γ* chain genes (*FGA*, *FGB* and *FGG*) as well as those of *FXIII* influence both total and γ’ fibrinogen concentrations, as well as clot properties [16]. What is unknown, however, is whether the relationship of alcohol with total or γ’ fibrinogen concentrations as well as the kinetics of clot formation, structural properties and fibrinolysis are modulated by SNPs. Knowledge of such interactions might improve our understanding of total and γ’ fibrinogen synthesis and regulation as well as give us more insight into clot formation, structure and lysis and could be used to modify risks by individualising preventive lifestyle approaches based on genetics.

Alcohol consumption can be measured by reported intake or through the use of biomarkers of liver function such as gamma (γ)-glutamyltransferase (GGT), aspartate aminotransferase (AST), alanine aminotransferase (ALT) and carbohydrate-deficient transferrin (CDT). GGT lacks specificity in that high levels could be associated with ageing [17–19], dyslipidaemia [20], atherosclerosis [21, 22], hypertension [21], diabetes [21], obesity [17] and/or liver damage [22]. The wide range of CDT measurements has resulted in a lack of uniform results and reference limits, hampering comparison of findings [23]. By mathematically combining these biomarkers, GGT–CDT, the diagnostic accuracy exceeds those of its parent components in detection of alcohol consumption [24–26]. To clarify the interrelationships of these factors, we conducted a study with the aim to establish, first, what associations there might be between total and γ’ fibrinogen concentration or fibrin plasma clot properties and alcohol intake/markers; and second, to determine whether there are interactions between these alcohol biomarkers and fourteen *fibrinogen* and two *FXIII* gene SNPs, that we identified from the literature to be of importance, in relation to total and γ’ fibrinogen concentrations and clot characteristics. Studying the relationship of alcohol and genetic variation on blood coagulation and fibrinolysis, by means of intake data and biomarkers, can increase our understanding of the mechanisms of how these factors influence haemostasis in individuals harbouring specific genetic variants. This new knowledge could ultimately improve prevention and intervention of CVD.

## Materials and methods

### Study design, population selection and ethics approval

This cross-sectional research is nested within the South African arm of the Prospective Urban and Rural Epidemiology (PURE) study [27]. A three-stage sampling process was followed based on power calculations of a previous study [28]. After obtaining gatekeeper permission, 6000 houses were randomly selected from urban and rural strata. The head of each family unit gave voluntary written informed consent before completing an interviewer-based questionnaire used to screen for eligibility. From the households, 4000 individuals with no reported use of chronic medication (excluding blood pressure medication) and/or any self-reported acute illness were identified. During the baseline period, biological samples were collected from 1006 rural and 1004 urban, apparently healthy African men and women who provided written informed consent. The study protocol was approved by the Ethics Committee of the authors’ university (NWU-00016-10-A1 and NWU-00034-17-A1–02) honouring the Declaration of Helsinki of 1975 and 2000.

### Lifestyle factor assessment

Questionnaires were interviewer-based in the participants’ home language and provided details regarding demographic and lifestyle factors, including tobacco. The physical activity index questionnaire, developed and validated locally, was completed [29]. Amounts of foods and beverages including alcohol consumed over the previous month were determined by a culture-sensitive, validated quantitative food frequency questionnaire (QFFQ) [30, 31] computerised using the *FoodFinder3®* programme based on the South African Food Composition Tables. Beer, homemade brews, spirits and wine were considered to contain 3.6 g, 3 g, 36 g and 9.4 g of pure alcohol per 100 g, respectively.

### Anthropometrical and blood pressure assessment

Participants’ weight and height were measured to calculate body mass index (BMI; kg/m^2^). Waist circumference was measured at the narrowest point between the 10th rib and iliac crest. Blood pressure – systolic and diastolic – was measured with the OMRON HEM-757 apparatus (Omron Healthcare, Kyoto, Japan) with the cuff on the left arm.

### Blood collection, sampling and storage

Fasting blood samples were collected between 07:00 and 11:00. Citrate-treated (3.8%) whole blood was centrifuged at 2000 x *g* for 15 min to yield buffy coat for DNA isolation and plasma specimens for coagulation markers. Ethylenediamine tetra-acetic acid-treated whole blood was used for glycated haemoglobin A1c (HbA1c) analysis. Coagulated blood was collected to yield serum for lipids, C-reactive protein (CRP), GGT, %CDT, ALT, and AST analysis. Aliquots were frozen on dry ice, stored in the field at − 18 °C and then, after 2–4 days, at − 80 °C until analysed.

### Biochemical analyses

Serum lipids [i.e. total cholesterol (TC), highdensity lipoprotein cholesterol (HDL-C) and triglycerides], high sensitivity CRP, GGT, ALT and AST concentrations were assayed using a Sequential Multiple Analyser (Konelab 20i, Thermo Fisher Scientific Oy, Vantaa, Finland). Low-density lipoprotein cholesterol (LDL-C) was calculated using the Friedewald formula. HbA1c concentrations were determined using the D-10 Haemoglobin testing system (Bio-Rad Laboratories, Hercules, CA, USA). Serum %CDT was quantified using an in vitro heterogeneous immunoassay with column separation followed by turbidimetric measurements (Axis-Shield %CDT kit, Oslo, Norway) with a measuring range of 1.5–24 mg/L of transferrin and a cut-off value of 2.6%. We used the following formula: 0.8 x ln (GGT) + 1.3 x ln(%CDT) to calculate GGT-CDT [25, 26, 32]. Fibrinogen was determined using the adapted Clauss method (Multifibrin U-test, BCS coagulation analyser, Dade Behring, Deerfield, IL, USA). Fibrinogen γ’ was quantified with an ELISA using a 2.G2.H9 mouse monoclonal coating antibody against the human γ’ sequence (Santa Cruz Biotechnology, Santa Cruz, USA) for antigen capture and a goat polyclonal antibody against human fibrinogen (Antibody 7539, Abcam for development, Cambridge, USA) [33, 34]. To obtain plasma fibrinolytic potential, reported as CLT, turbidimetric analysis (A405 nm) was used [35] with modified tissue factor and tissue-plasminogen activator (tPA) concentrations to attain CLTs of 60 min. Final concentrations were: tissue factor (125x diluted – an estimated final concentration of 59 pM; Dade Innovin, Siemens Healthcare Diagnostics Inc., Marburg, Germany), CaCl_2_ (17 mmol/L), tPA (100 ng/mL; Actilyse, Boehringer Ingelheim, Ingelheim, Germany) and phospholipid vesicles (10 μmol/L; Rossix, Mölndal, Sweden). Kinetics of clot formation (lag time and slope), and structural clot properties (maximum absorbance) were calculated from the turbidity curves [33]. Lag time represents the time required for fibrin fibres to grow to sufficient length to allow lateral aggregation as well as activation of the coagulation cascade. The slope represents the rate of lateral aggregation and maximum absorbance indicates clot density. The coefficient of variation for all assays was < 10%. Using whole blood, a rapid first response HIV card test 1–2.0 (Transnational Technologies Inc. PMC Medical, Nani Daman, India) was done and the outcome confirmed with a Pareeshak test (BHAT Bio-tech, Bangalore, India).

### DNA extraction, sequencing and genotyping

Genomic DNA was extracted using FlexiGene™ (QIAGEN, catalogue number 51206) and the Maxwell® 16 kits. *FGA* 2224G/A (rs2070011), *FGA* 6534A/G (rs6050), *FGB* 1038G/A (rs1800791), *FGB* Arg448Lys, G/A (rs4220), *FGB* –148C/T (rs1800787), *FGG* 10034C/T (rs2066865), *FGG* 9340 T/C (rs1049636), the F*XIII* His95Arg A/G (rs6003) and *FXIII* Val34Leu, C/A (rs5985) were genotyped as described elsewhere [36].

Downstream quality control included a check for individuals’ successfully genotyped (missingness), minor allele frequencies (MAF) and a tally of Mendelian error via Haploview (v4.2; http://www.broad.mit.edu/mpg/haploview) and PLINK (v1.07; http://pngu.mgh.harvard.edu/purcell/plink/) software. Pairwise linkage-disequilibrium (LD) between the SNPs have been reported previously [36]. Because three SNPs (rs7439150, rs1800789 and rs1800787) were not in complete LD, they are reported separately.

### Statistical analysis

*Statistica®* version 13.3 (TIBCO Software Inc., Tulsa, Oklahoma, USA) and SAS System for Windows (SAS Institute Inc., Cary, NC, USA) were used for analyses. Normally distributed data are expressed as mean (95% confidence intervals (CI)), and not-normally distributed data as median (lower and upper quartiles). Differences between the genders, HIV status and drinking status in terms of coagulation factors were determined by Mann–Whitney U tests and among tobacco use subgroups, using Kruskal–Wallis analysis of variance (ANOVA). Spearman correlations were performed to test for statistical dependence between variables and to identify confounders and/or co-variates for inclusion in subsequent analysis (LDL-C, age, HbA1c, gender, HIV status, tobacco use, BMI). Spearman partial correlations were conducted adjusting for fibrinogen to determine whether the relationship between alcohol intake or its markers and clot properties are influenced by fibrinogen concentration additionally when CLT was the outcome we adjusted for fibrinogen, HbA1c and BMI.

To investigate whether markers of alcohol consumption have interactive effects with the fibrinogen and *FXIII* gene polymorphisms in predicting total and %γ’ fibrinogen concentration and clot properties, general linear models (adjusting for age, gender, HIV status, tobacco use, LDL-c, HbA1c and BMI) with interactions were performed. Interactions that remained after accounting for multiple testing and false discovery rates according to Hochberg and Benjamini are reported; the statistical threshold for significance was *p* < 0.004 when the false discovery rate was set at 25% [37]. To describe the interaction, Spearman partial correlations adjusting for LDL-C, age, HbA1c, gender, HIV status, tobacco use and BMI and additionally for fibrinogen, when the interaction was in relation to maximum absorbance, were used. 

## Results

### Participants characteristics and correlations between measured variables and total fibrinogen, %γ’ fibrinogen concentration and plasma clot properties

Participant characteristics, 44.4% being drinkers and 55.6% abstainers, including data on alcohol intake and its biomarkers, are presented in Table 1. Markers of alcohol intake differed significantly between alcohol consumers and abstainers. The median alcohol intake of 659 participants who drank was 15.4 (6.43–34.7) g/day. Total fibrinogen (*p* < 0.0001), maximum absorbance (*p* < 0.0001), and CLT (*p* < 0.01) were higher in female participants. In HIV infected participants, a lower fibrinogen (*p* < 0.0001), slope (*p* < 0.0001) and maximum absorbance (*p* < 0.0001), but higher %γ’ fibrinogen (*p* < 0.0001) were observed. No differences were detected in haemostatic markers among the subgroups for tobacco use, except CLT (*p* < 0.0001) which was longer in the non-smokers.

**Table 1:** Selected characteristics of the study participants

Median (interquartile range) or (n: n)					
		Whole group	Abstainers	Drinkers	Differences between abstainers and drinkers (***p*** value)
**Gender (Men: Women)#**		747: 1263	259: 818	465: 407	< 0.001
**Age (yr)**		48.0 (41.0–56.0)	48.5 (41.5–57.5)	48.0 (42.1–55.4)	0.20
**Blood pressure (mmHg)**					
Diastolic		87.0 (78.0–97.0)	85.0 (76.0–95.0)	89.0 (79.0–99.0)	< 0.0001
Systolic		130 (116–147)	127 (114–144)	133 (120–150)	< 0.0001
**Tobacco use (%)**					
Never (1)		52.7	4.3	3.5	< 0.0001
Former (2)		4.14	38.3	69.6	
Current (3)		42.7	57.4	26.9	
**PA index**		7.33 (5.79–8.67)	7.70 (6.00–8.80)	6.80 (5.50–8.40)	< 0.0001
**BMI (kg/m**^**2**^**)**		22.9 (19.3–28.6)	25.1 (20.8–31.0)	20.9 (18.4–24.9)	< 0.001
**Waist circumference (cm)**		77.5 (70.2–87.7)	81.0 (71.6–90.3)	74.4 (69.3–83.2)	< 0.0001
**Biochemical markers**	**HIV status (Unaffected n: Affected n)#**	1668: 326	917: 157	707: 156	0.04
	**TC (mmol/L)**	4.82 (4.01–5.87)	4.93 (4.11–6.05)	4.74 (3.95–5.67)	0.001
	**LDL-C (mmol/L)**	2.79 (2.08–3.65)	3.02 (2.25–3.88)	2.56 (1.87–3.25)	< 0.0001
	**HDL-C (mmol/L)**	1.42 (1.06–1.87)	1.32 (1.02–1.67)	1.60 (1.14–2.09)	< 0.0001
	**Triglycerides (mmol/L)**	1.08 (0.82–1.55)	1.12 (0.82–1.56)	1.30 (0.36–7.88)	0.08
	**HbA1c (%)**	5.50 (5.30–5.80)	5.60 (5.30–5.90)	5.50 (5.10–5.80)	< 0.0001
	**CRP (mg/L)**	3.29 (0.96–9.34)	3.40 (0.89–9.44)	3.04 (1.04–9.13)	0.68
	**Total fibrinogen (g/l)**	2.90 (2.30–5.00)	3.10 (2.40–5.50)	2.70 (2.10–4.30)	< 0.0001
	**Fibrinogen γ’ (%)**	10.2 (7.14–14.6)	10.3 (7.12–14.7)	10.1 (7.34–14.6)	0.81
	**Lag time (min)**	6.52 (5.10–7.78)	6.50 (5.13–7.77)	6.58 (5.10–7.78)	0.96
	**Slope (×10**^**−03**^ **au/s)**	8.90 (6.49–11.9)	9.23 (6.72–12.3)	8.52 (6.15–11.6)	< 0.001
	**Maximum absorbance (nm)**	0.42 (0.33–0.52)	0.43 (0.35–0.53)	0.40 (0.31–0.49)	< 0.001
	**CLT (min)**	57.2.(50.9–63.8)	59.2 (53.8–66.0)	52.3 (47.5–60.5)	< 0.001
	**GGT (U/l)**	46.0 (29.7–88.0)	36.4 (26.3–55.0)	73.0 (40.1–157)	< 0.001
	**%CDT**	2.67 (1.97–3.59)	2.35 (1.80–3.08)	3.07 (2.30–4.32)	< 0.001
	**GGT-CDT**	4.37 (3.76–5.12)	3.98 (3.54–4.52)	4.99 (4.29–5.72)	< 0.001
	**ALT (IU/L)**	17.1 (12.0–25.1)	16.0 (11.6–21.8)	19.5 (13.0–29.1)	< 0.001
	**AST (IU/L)**	26.0 (19.2–39.0)	23.2 (18.0–31.0)	31.7 (22.0–52.1)	< 0.001
**Dietary intake**	**Alcohol intake of consumers (g/day); abstainers: drinkers (n:n)#**	15.4 (6.43–34.7); 824: 659	–	15.4 (6.43–34.7)	–
	**Energy (kJ)**	7175 (5268–10,001)	6508 (4834–8763)	8077 (5953–11,792)	< 0.001
	**Protein (%TE)**	11.6 (10.4–12.9)	11.6 (10.3–12.8)	11.6 (10.5–13.0)	0.13
	**Carbohydrate (%TE)**	60.3 (54.2–67.5)	63.1 (56.2–69.6)	57.5 (52.4–63.3)	< 0.001
	**Total fat (%TE)**	22.5 (17.4–27.7)	22.7 (18.1–28.2)	22.4 (16.6–27.1)	< 0.001

*ALT* Alanine transaminase, *AST* Aspartate aminotransferase, *BMI* Body mass index, *CLT* Clot lysis time, *CRP* High sensitivity C-reactive protein, *%CDT* Percentage carbohydrate deficient transferrin, *GGT* Gamma-glutamyltransferase, *GGT-CDT* 0.8 x ln(GGT) + 1.3 x ln(%CDT), *HbA1c* Glycosylated haemoglobin A1c, *HDL-C* High-density lipoprotein cholesterol, *HIV* Human immunodeficiency virus, *LDL-C* Low-density lipoprotein cholesterol; n, number of patients, *PA* Physical activity, *PAI-1*_*act*_ Plasminogen activator inhibitor type-1 activity, *TC* Total cholesterol, *TE* Total energy

#Total sample is 2010, but numbers are slightly different for several variables including alcohol intake due to missing data

Positive correlations were found between self-reported alcohol intake and %CDT (r = 0.43), GGT (r = 0.32), ALT (r = 0.20) and AST (r = 0.33), with the combined marker, GGT-CDT, demonstrating the strongest correlation (r = 0.50). In Table 1 we present differences in total fibrinogen and kinetics of clot formation between drinkers and abstainers and in Table 2 we present correlations of alcohol intake and its markers with total and γ’ fibrinogen concentrations and clot properties. Total fibrinogen was lower, slope shorter, clot density less and CLT shorter in drinkers than abstainers. All alcohol intake markers correlated negatively with total fibrinogen (with AST having the strongest correlation) and CLT (with GGT–CDT having the strongest correlation). AST was the only alcohol intake marker to correlate with %γ’ fibrinogen and, albeit weakly, with lag time (both negative correlations). Slope correlated negatively with GGT (albeit weakly), ALT and AST. All alcohol biomarkers except %CDT correlated negatively with maximum absorbance.

**Table 2:** Correlations (r) of participant characteristics with total and γ’ fibrinogen concentrations and clot properties

Participant’s characteristics (Whole group)		Total fibrinogen	% fibrinogen γ’	Lag time	Slope	Maximum absorbance	CLT
**Age (yr)**		0.16**	−0.11***	0.13***	0.11***	0.15***	−0.003
**PA index**		0.01	− 0.06*	− 0.20***	0.17***	− 0.06*	0.04
**BMI (kg/m**^**2**^**)**		0.16***	0.06*	−0.006	0.03	0.12***	0.48**
**Waist circumference (cm)**		0.14***	0.06*	−0.004	0.02	0.10***	0.42**
**Biochemical markers**	**TC (mmol/L)**	0.08***	−0.07**	0.03	0.03	0.06*	0.09***
	**LDL-C (mmol/L)**	0.13***	−0.03	0.03	0.06**	0.09***	0.16***
	**HDL-C (mmol/L)**	−0.08***	− 0.13***	0.04	0.03	−0.01	− 0.19***
	**Triglycerides (mmol/L)**	0.07**	0.03	−0.03	−0.07**	0.004	0.20***
	**HbA1c (%)**	0.20***	0.008	0.008	0.07**	0.12***	0.20***
	**CRP (mg/L)**	0.42***	−0.07**	0.04	0.22***	0.42**	0.23***
	**GGT (U/l)**	−0.10***	− 0.01	0.005; − 0.03	−0.07**; − 0.01	−0.09***; − 0.05*	−0.15***; − 0.19*** − 0.134***
	**%CDT**	− 0.09***	− 0.01	0.006; − 0.009	0.03; 0.09**	− 0.04; 0.02	− 0.14***; − 0.25*** − 0.111***
	**GGT-CDT**	− 0.14***	− 0.007	− 0.02; − 0.02	− 0.04; 0.03	− 0.13***; − 0.04	− 0.30***; − 0.28*** − 0.162***
	**ALT (IU/L)**	− 0.11***	0.02	−0.03; − 0.02	−0.11***; − 0.07**	− 0.14***; − 0.10***	−0.01***; − 0.07** − 0.048*
	**AST (IU/L)**	−0.22***	0.11***	−0.04*; − 0.03	−0.12***; − 0.04*	−0.18***; − 0.01*	−0.24***; − 0.21*** − 0.107***
**Alcohol intake (g/day)**^a^		−0.09**	− 0.08*	− 0.06; − 0.05*	0.04; 0.03	− 0.08*; − 0.05*	−0.18***; − 0.24** − 0.145***

*ALT* Alanine transaminase, *AST* Aspartate aminotransferase, *BMI* Body mass index, *CLT* Clot lysis time, *CRP* High sensitivity C-reactive protein, *%CDT* Percentage carbohydrate deficient transferrin, *GGT* Gamma-glutamyltransferase, *GGT-CDT* 0.8 x ln(GGT) + 1.3 x ln(%CDT), *HbA1c* Glycosylated haemoglobin A1c, *HDL-c* High-density lipoprotein cholesterol, *LDL-c* Low-density lipoprotein cholesterol, *n* Number of patients, *PA* Physical activity, *r* Correlation coefficient, *TC* Total cholesterol

First r-value is for Spearman correlation; second for Spearman partial correlations of alcohol markers with clot properties adjusting for fibrinogen to determine how alcohol associates with clot properties independent of fibrinogen concentrations; third r-value for Spearman partial correlations of alcohol markers with CLT adjusting for fibrinogen, HbA1c and BMI to determine how alcohol associates with CLT independent of fibrinogen concentrations as well as HbA1c and BMI

*p < 0.05; **p ≤ 0.01; ***p ≤ 0.001; ^a^only consumers included

Variables (age, BMI and HbA1c) that were associated with the haemostatic markers or subgroups across which haemostatic variables differed (gender, HIV status and tobacco use subgroups), were treated as covariates within subsequent analyses. By adjusting for HbA1c we also accounted for non-alcoholic fatty liver disease (NAFLD), because NAFLD and type 2 diabetes mellitus generally coexist [38, 39]. We present the correlations for the drinkers and abstainers separately in Table 3. The correlations remained largely the same except that, in drinkers, fibrinogen and maximum absorbance did not appear to increase with BMI and the positive association of %γ’ fibrinogen with AST remained only in abstainers.

**Table 3:** Correlations (r) stratified for drinkers and abstainers

Participant characteristics		Total fibrinogen		%fibrinogen γ’		Lag time		Slope		Maximum absorbance		CLT	
		Drinkers	Abstainers	Drinkers	Abstainers	Drinkers	Abstainers	Drinkers	Abstainers	Drinkers	Abstainers	Drinkers	Abstainers
**Age (yr)**		0.19***	0.15***	−0.14***	−0.11***	0.05	0.05*	0.15***	0.11***	0.19***	0.18***	−0.12***	0.02
**PA index**		−0.02	0.004	−0.02	− 0.05*	− 0.24***	− 0.20***	0.20***	0.17***	−0.04	− 0.07**	0.04	0.03
**BMI (kg/m**^**2**^**)**		0.09*	0.17***	0.10**	0.05*	−0.05	− 0.03	− 0.03	0.03	0.05	0.15***	0.48**	0.48**
**Waist circumference (cm)**		0.06	0.14***	0.08*	0.06*	−0.08*	− 0.04	− 0.006	0.02	0.03	0.11***	0.40***	0.43**
**Biochemical markers**	**TC (mmol/L)**	0.06	0.07**	− 0.11**	− 0.07**	− 0.02	0.006	0.05	0.03	0.07*	0.10***	0.11**	0.16***
	**LDL-C (mmol/L)**	0.08*	0.12***	−0.02	− 0.03	0.02	0.03	0.06	0.06*	0.10**	0.14***	0.25***	0.28**
	**HDL-C (mmol/L)**	−0.01	− 0.09***	− 0.23***	− 0.12***	− 0.01	0.03	0.10**	0.03	0.03	− 0.01	− 0.36***	− 0.31**
	**Triglycerides (mmol/L)**	0.02	0.07**	0.06	0.03	−0.09*	− 0.08***	− 0.09*	−0.07**	− 0.04	−0.005	0.35***	0.33**
	**HbA1c (%)**	0.17***	0.20***	0.002	0.007	0.03	− 0.02	0.05	0.08**	0.18***	0.18***	0.28***	0.35**
	**CRP (mg/L)**	0.39***	0.42**	−0.06	− 0.06*	0.01	0.04	0.23***	0.22***	0.40**	0.42**	0.20***	0.23***
	**GGT (U/l)**	−0.15***	−0.11***	− 0.03	− 0.004	− 0.07*; − 0.07	−0.03; − 0.01	−0.05; 0.04	− 0.07**; − 0.03	−0.20***; − 0.11**	−0.11***; 0.04	− 0.23***; − 0.21*** − 0,182***	−0.21***; 0.01 − 0,021
	**%CDT**	−0.02	− 0.10***	− 0.10**	− 0.01	0.02; 0.02	− 0.01; − 0.05	0.06; 0.07	0.03; 0.13***	0.005; 0.05	− 0.06*; 0.02	− 0.27***; − 0.29*** − 0,179***	−0.26***; − 0.09* − 0,001
	**GGT-CDT**	−0.13***	− 0.15***	− 0.08*	− 0.001	− 0.03; − 0.02	− 0.02; − 0.05	−0.009; 0.06	− 0.04; 0.07	−0.15***; − 0.05	−0.13***; 0.03	− 0.34***; − 0.33*** − 0,249***	−0.31**; − 0.07 − 0,026
	**ALT (IU/L)**	− 0.14***	− 0.12***	0.01	0.02	−0.06; − 0.05	−0.02; 0.013	− 0.07*; − 0.00	−0.12***; − 0.13***	−0.20***; − 0.11**	−0.14***; − 0.05	−0.11**; − 0.09* − 0,097*	−0.10***; 0.028 0,01
	**AST (IU/L)**	−0.21***	−0.23***	0.06	0.11***	−0.06; − 0.04	−0.04; − 0.04	−0.08*; − 0.01	−0.12***; − 0.06	−0.21***; − 0.1*	−0.18***; − 0.02	−0.25***; − 0.22*** − 0,15***	−0.24***; − 0.094** − 0,038
**Alcohol intake (g/day)**		−0.09**	–	− 0.08*	–	−0.06; − 0.04	–	0.04; 0.08*	–	−0.08*; − 0.02	–	− 0.18***; − 0.19*** − 0,137***	–

First r-value is for Spearman correlation; second r-value for Spearman partial correlations of alcohol markers with clot properties adjusting for fibrinogen to determine how alcohol associates with clot properties independent of fibrinogen concentrations; third r-value for Spearman partial correlations of alcohol markers with CLT adjusting for fibrinogen, HbA1c and BMI to determine how alcohol associates with CLT independent of fibrinogen concentrations as well as HbA1c and BMI

*p < 0.05; **p ≤ 0.01; ***p ≤ 0.001

*ALT* Alanine transaminase, *AST* Aspartate aminotransferase, *BMI* Body mass index, *CLT* Clot lysis time, *CRP* High sensitivity C-reactive protein, *%CDT* Percentage carbohydrate deficient transferrin, *GGT* Gamma-glutamyltransferase, *GGT-CDT* 0.8 x ln(GGT) + 1.3 x ln(%CDT), *HbA1c* Glycosylated haemoglobin A1c, *HDL-c* High-density lipoprotein cholesterol, *LDL-c* Low-density lipoprotein cholesterol, *n* Number of patients, *PA* Physical activity, *r* Correlation coefficient; *TC* Total cholesterol

When partial correlations adjusting for fibrinogen were conducted to determine whether the relationship between alcohol intake or its markers and clot properties are influenced by the fibrinogen concentration, the associations with lag time and slope were no longer noteworthy. Of the relationships with maximum absorbance, the ones with %CDT and GGT-CDT did not remain after accounting for total fibrinogen. The associations between alcohol intake and its markers with CLT remained after controlling for total fibrinogen concentration, particularly in drinkers, but not in abstainers. These associations also remained after additional adjustment for BMI and HbA1c (Tables 2 and 3).

### Gene-alcohol interactions

Detailed results of the individual fibrinogen SNPs and their associations with fibrinogen concentration and clot properties have been described [36]. Here we focus on the combined effects of markers of alcohol consumption and genetics of *fibrinogen* and *FXIII* in relation to total fibrinogen, %γ’ fibrinogen and clot properties. Table 4 reports interactions at *p* < 0.004 after full adjustment.

**Table 4:** *FGB*, *FGA*, *FGG* and *FXIII* gene polymorphisms’ interactions with alcohol intake/markers in relation to haemostasis

Interaction	Interaction ***p***-value	Genotype	r	***p***-value	Slope (y = mx + c)	CI of slope
**QFFQ alcohol intake interactions in relation to maximum absorbance**						
***FGB*: rs7439150**	< 0.001	AA AG GG	−0.24 − 0.02 − 0.08	0.65 0.83 0.003	−0.008 − 0.0004 − 0.0004	−0.004; 0.02 − 0.002; 0.0001 − 0.001; 0
***FGB*: rs1800790**	< 0.001	GG GA AA	− 0.08 0.18 ^a^	0.002 0.09 ^a^	− 0.0004 0.004 ^a^	−0.001; 0 0.002; 0.006 ^a^
***FGG*: rs1049636**	0.001	TT TC CC	−0.11 0.05 − 0.16	< 0.001 0.31 0.33	− 0.0006 0.0007 − 0.001	−0; 0.001 0; 0.001 − 0.003; 0.001
**ALT interactions in relation to total fibrinogen**						
***FGB*: rs1800791**	0.001	GG GA AA	−0.10 0.02 0.93	< 0.001 0.80 0.07	−0.008 0.01 − 0.005	−0.02; − 0.002 0; 0.02 − 0.27; 0.25
***FGA*: rs6050**	0.003	AA AG GG	− 0.07 − 0.08 − 0.26	0.08 0.05 0.002	0.006 − 0.01 − 0.03	−0.003; 0.02 − 0.02; − 0.003 − 0.05; − 0.007
***FGG*: rs2066865**	0.004	CC CT TT	−0.06 − 0.12 − 0.27	0.12 0.01 0.004	0.005 − 0.01 − 0.03	− 0.003; 0.01 − 0.02; − 0.003 − 0.05; − 0.001
**ALT interactions in relation to maximum absorbance**						
***FGB*: rs1800791**	< 0.001	GG GA AA	−0.15 0.01 − 0.65	< 0.001 0.86 0.35	−0.001 0.0009 − 0.005	−0.001; − 0.001 0; 0.002 − 0.04; 0.03
***FGG*: rs1049636**	0.003	TT TC CC	− 0.18 − 0.04 − 0.16	< 0.001 0.50 0.33	−0.001 0.0006 − 0.002	−0.002; − 0.001 0; 0.001 − 0.005; 0.001
***FXIII*: rs6003**	< 0.001	AA AG GG	−0.04 − 0.16 − 0.14	< 0.001 < 0.001 0.64	0.002 − 0.0008 − 0.001	0; 0.003 − 0.001; 0 − 0.002; − 0.001
**AST interactions in relation to maximum absorbance**						
***FGB*: rs7439150**	< 0.001	AA AG GG	−0.13 − 0.16 0.91	< 0.001 0.03 0.01	−0.0003 − 0.0003 0.0	−0.001; 0 − 0.001; 0 − 0.008; 0.04
**GGT–CDT interactions in relation to total fibrinogen**						
***FGB*: rs2227388**	0.001	AA AG GG	−0.06 − 0.03 − 0.51	0.03 0.55 0.001	−0.11 − 0.16 − 3.14	−0.23; 0.003 − 0.49; 0.18 − 14.9; 8.63

*A* Adenine, *ALT* Alanine transaminase, *AST* Aspartate aminotransferase, *C* Cytosine, *CDT* Carbohydrate deficient transferrin, *FGA* Fibrinogen alpha chain, *FGB* Fibrinogen beta chain, *FGG* Fibrinogen gamma chain, *G* Guanine, *GGT* Gamma-glutamyltransferase, *GGT-CDT* 0.8 x ln(GGT) + 1.3 x ln(%CDT), *QFFQ* Quantitative food frequency questionnaire, *rs* Reference sequence, *T* Thymine

^a^Stratification according to rs1800790 resulted in too few individuals to perform analysis

Self-reported alcohol intake interacted with two *FGB* (rs7439150 and rs1800790) and one *FGG* (rs1049636) SNP in relation to maximum absorbance. ALT interacted with *FGB*: rs1800791, *FGA*: rs6050 and *FGG*: rs2066865 in relation to total fibrinogen with the inverse relationship being strongest in those homozygous for the minor alleles, and *FGB*: rs1800791, *FGG*: rs1049636 and *FXIII*: rs6003 in relation to maximum absorbance. AST interacted with *FGB*: rs7439150 also in relation to maximum absorbance. GGT–CDT modulated one *FGB* SNP: rs2227388 in relation to total fibrinogen only with this inverse relationship being strongest in those homozygous for the minor A allele. The modulatory influence on maximum absorbance and fibrinogen concentration seems to be strongest in the minor allele carriers in most cases, except for alcohol intake at rs1049636, ALT at rs1049636 and rs6003, AST at rs7439150 and GGT-CDT at rs2227388, respectively. Additional adjustment for total fibrinogen in the interactions observed between the SNPs and maximum absorbance did not change their statistical significance.

## Discussion

Alcoholism is a notorious global public health issue, which calls for evidence-based recommendations – which have been much disputed – regarding the pros and cons of alcohol consumption. Whereas its harmful effects are well known and can be devastating, in some circumstances alcohol can confer benefits. We report here how alcohol is correlated advantageously with biomarkers of blood clotting such as total and γ’ fibrinogen, as well as certain clotting properties by comparing drinkers with abstainers. More specifically, from a genetics perspective, alcohol modulated the influence of *fibrinogen* SNPs on total fibrinogen concentrations and the *fibrinogen* SNPs as well as the *FXIII* SNP (rs6003) on clot density. This implies that those harbouring specific genetic variants could benefit more than others in respect of alcohol consumption in their susceptibility to coagulation and fibrinolysis.

Among alcohol consumers, the median intake was 15.4 (6.43–34.7) g/day [40]. Self-reported alcohol intake correlated positively with all its biochemical markers. The strongest correlation was with GGT-CDT, which supports the claim of its superior diagnostic accuracy [26]. Alcohol intake and its markers correlated negatively, albeit moderately, with total fibrinogen, as well as with most of the clot properties, although after additional adjustment for fibrinogen, significance remained for CLT only. The fact that these associations were consistently found for both self-reported intake as well as biomarkers, suggests that although the relationship is only modest, it is likely not a spurious finding. Correlation between alcohol biomarkers for drinkers and abstainers were largely the same. However, in drinkers, fibrinogen and maximum absorbance do not appear to increase with BMI as is seen in non-drinkers, potentially due to a protective effect of alcohol. Percentage γ’ fibrinogen was positively associated with AST and inversely with alcohol intake; however, when stratifying for consumption, negative associations were also observed for %CDT, and GGT-CDT in the drinkers and the positive association with AST was present in the abstainers only, suggesting that this relationship is likely not driven by alcohol consumption.

Our data are furthermore, in agreement with conclusions drawn by a number of comprehensive reviews and a meta-analysis on the topic [12–14]. The inverse relationship between fibrinogen concentrations and alcohol consumption is attributed mainly to ethanol [12], but the possibility of the result being from the ingredients accompanying ethanol in alcoholic beverage, or to their synergistic effect, is plausible. Early animal studies suggested that ethanol may interfere with hepatic plasma protein synthesis [41]. Exposure of hepatoma cells to ethanol diminished fibrinogen production by 18–20% by decreasing the transcription of fibrinogen genes [42]. Both fibrinogen [43–45] and the markers of alcohol intake, are synthesised primarily by the liver [46]. Because alcohol has direct effects on the liver, the relationship between fibrinogen and liver enzymes is expected. Our report is one of the first to investigate γ’ fibrinogen in relation to alcohol consumption and to find an inverse relationship. Increased levels of γ’ fibrinogen have been related to denser blood clots resistant to lysis [33, 47, 48], thus reduced levels may provide a mechanistic pathway through which alcohol consumption can improve clot structure.

We also observed notable associations between clot properties (slope, density and lysability) and alcohol consumption. Because fibrinogen is one of the most important factors influencing clot characteristics [49], its elevated levels can result in increased clot density [33, 50, 51]. Moderate alcohol intake may therefore, also decrease clot density, due, at least in part, to the inhibitory effect thereof on fibrinogen concentration. Because several relationships between alcohol intake and clot properties remained after adjustment for fibrinogen concentrations, this could indicate that the relationships were not only due to the associated lower fibrinogen concentrations, but to potential direct effects of alcohol intake on these clot properties, especially CLT. It is possible that alcohol affects CLT, through other mechanisms than clot structure. Plasminogen activator inhibitor type-1 activity [52, 53], thrombin activatable fibrinolysis inhibitor [54–56], tPA [53], BMI [52, 57, 58], HbA1c [52], triglycerides [57], blood pressure [57] and CRP [57] have all been reported to affect CLT. In our study some of the strongest correlations of CLT were with BMI, triglycerides and HbA1c. Of the latter, BMI and HbA1c correlated negatively with alcohol intake and its markers whereas triglycerides correlated positively with some (ALT, AST and GGT), but negatively with others (%CDT and alcohol intake - results not shown). When additionally adjusting for BMI and HbA1c, the negative association between alcohol and markers thereof with CLT remained. This suggests that the negative association between alcohol consumption and CLT is only partly due to the decreased fibrinogen concentration, BMI and HbA1c and the exact mechanisms remain to be clarified.

To our knowledge, this study is the first of its kind to investigate the associations of alcohol intake and its markers in the presence of candidate *fibrinogen* and *FXIII* genotypes on total fibrinogen, %γ’ fibrinogen and clot properties. A genome wide association study failed to detect any gene-alcohol interactions in relation to haemostasis [13]. We revealed that the relationship of certain SNPs with fibrinogen concentration and clot properties, in particular clot density, are modulated by alcohol intake/markers, and that the relationship with clot density remained after adjustment for fibrinogen, indicating that this effect is at least partly independent of fibrinogen concentration. Genetic variation in the fibrinogen genes may alter the magnitude of fibrinogen expression in response to alcohol intake. Future research should investigate the mechanisms behind the interactions we observed per loci. In this respect, we know that alcohol has the ability to perturb normal patterns of DNA methylation [59] impacting epigenetic regulatory mechanisms [60]. Moreover, ethanol metabolites can bind to transcription factors and/or modify chromatin structure, thereby altering gene expression [59, 60].

Even though our report is one of the first to investigate a broad range of haemostatic markers in relation to alcohol consumption and to take genetic factors into consideration, we dealt with certain limitations. Using the QFFQ method could result in over- or under-reporting of alcohol consumption and cannot discern between binge or moderate drinkers. However, our findings are validated by also observing associations and interactions with biomarkers reflecting alcohol intake. While we included two *FXIII* SNPs, we could unfortunately not measure FXIII levels to provide supporting evidence for the effect of FXIII levels on fibrin clot structure. We believe that, even with these limitations and the observational nature of our study design, our results are reliable and of importance to better understand those lifestyle factors such as alcohol intake that predispose individuals to – or protect them from – unfavourable haemostatic factors that might ultimately lead to CVD.

## Conclusion

Some authorities insist that—even in small quantities—the harmful effects of alcohol outweigh the benefits, because alcohol-related liver disease remains the main cause of liver-related mortality worldwide [61]. Our research shows that, in terms of haemostasis, modest amounts of alcohol seem to be beneficial. Because fibrinogen and clot properties are closely related to cardiovascular risk – and limited alcohol consumption could result in minor reductions in fibrinogen levels and improvements in blood clot structure – this relationship is of potential clinical importance. From a genetics viewpoint, we show here that for individuals harbouring certain genotypes, alcohol intake may be more beneficial than for others. A better understanding of diet-gene interactions has important implications for therapeutic decision making and lifestyle education. Nevertheless, drinking alcohol should never be recommended to improve health status; and for current drinkers, the prevailing evidence supports the adoption of even lower limits of consumption than are presently in most guidelines.

## Data Availability

The datasets used and/or analysed during the current study are available from the Principle Investigator of the South African arm of the PURE-study, Prof. Iolanthé M. Kruger, on reasonable request.

## Abbreviations

ALT: Alanine aminotransferase
AST: Aspartate aminotransferase
BMI: Body mass index
CDT: Carbohydrate deficient transferrin
CVD: Cardiovascular disease
CLT: Clot lysis time
CRP: C-reactive protein
F: Factor
*FGA*, *FGB*, *FGG*: *Fibrinogen α*, *β* and *γ* chain genes
GGT: Gamma-glutamyltransferase
HbA1c: Glycated haemoglobin A1c
LD: Linkage-disequilibrium
LDL-C: Low-density lipoprotein cholesterol
MAF: Minor allele frequencies
NADH: Nicotinamide adenine dinucleotide
NAFLD: Non-alcoholic fatty liver disease
QFFQ: Quantitative food frequency questionnaire
SNPs: Single nucleotide polymorphisms
PURE: South African arm of the Prospective Urban and Rural Epidemiology study
PA: Physical activity
tPA: Tissue-plasminogen activator
TC: Total cholesterol
HDL-C: High-density lipoprotein cholesterol
